# Brain-wide map of efferent projections from rat barrel cortex

**DOI:** 10.3389/fninf.2014.00005

**Published:** 2014-02-05

**Authors:** Izabela M. Zakiewicz, Jan G. Bjaalie, Trygve B. Leergaard

**Affiliations:** Department of Anatomy, Institute of Basic Medical Sciences, University of OsloOslo, Norway

**Keywords:** anterograde transport, axonal tracing, brain atlas, connectivity, connectome, neuroanatomical tract tracing, neuroinformatics, wiring diagram

## Abstract

The somatotopically organized whisker barrel field of the rat primary somatosensory (S1) cortex is a commonly used model system for anatomical and physiological investigations of sensory processing. The neural connections of the barrel cortex have been extensively mapped. But most investigations have focused on connections to limited regions of the brain, and overviews in the literature of the connections across the brain thus build on a range of material from different laboratories, presented in numerous publications. Furthermore, given the limitations of the conventional journal article format, analyses and interpretations are hampered by lack of access to the underlying experimental data. New opportunities for analyses have emerged with the recent release of an online resource of experimental data consisting of collections of high-resolution images from 6 experiments in which anterograde tracers were injected in S1 whisker or forelimb representations. Building on this material, we have conducted a detailed analysis of the brain wide distribution of the efferent projections of the rat barrel cortex. We compare our findings with the available literature and reports accumulated in the Brain Architecture Management System (BAMS_2_) database. We report well-known and less known intracortical and subcortical projections of the barrel cortex, as well as distinct differences between S1 whisker and forelimb related projections. Our results correspond well with recently published overviews, but provide additional information about relative differences among S1 projection targets. Our approach demonstrates how collections of shared experimental image data are suitable for brain-wide analysis and interpretation of connectivity mapping data.

## Introduction

The characteristic grid-like arrangement of mystacial representations in the whisker barrel field of the primary somatosensory cortex (S1; Welker, 1971; Chapin and Lin, 1984; Dawson and Killackey, 1987; Welker et al., 1988; Fabri and Burton, 1991a) has made the rat barrel cortex a common model for anatomical and physiological investigations of sensory processing and brain plasticity (Petersen, 2007; Alloway, 2008; Wiest et al., 2008; Feldmeyer et al., 2013). The intracortical and subcortical connections of the S1 barrel cortex have been extensively mapped by use of axonal tract tracing and electrophysiological techniques, and many of the connections target brain regions involved in synchronization of body movements in reply to sensory stimuli (Alloway, 2008; Wiest et al., 2008). A considerable number of studies have shown that the S1 barrel cortex projects to the motor cortex (Chapin and Lin, 1984; Reep et al., 1990; Fabri and Burton, 1991a; Smith and Alloway, 2013), primary and secondary somatosensory cortex (Chapin and Lin, 1984; Koralek et al., 1990; Fabri and Burton, 1991a), insular cortex (Fabri and Burton, 1991a), perirhinal and ectorhinal cortex (Fabri and Burton, 1991a; Naber et al., 2000), auditory and visual cortex (Frostig et al., 2008; Sieben et al., 2013) while subcortical projections terminate bilaterally in the dorsal striatum (Brown et al., 1998; Alloway et al., 1999; Hoffer et al., 2005), ipsilaterally in the thalamus (Fabri and Burton, 1991b; Landisman and Connors, 2007), red nucleus (Ebrahimi-Gaillard and Roger, 1993), superior colliculus (Wise and Jones, 1977a; Hoffer et al., 2005), and pontine nuclei (Mihailoff et al., 1978; Wiesendanger and Wiesendanger, 1982; Mihailoff et al., 1985; Leergaard and Bjaalie, 2007), and contralaterally in the trigeminal nuclei (Killackey et al., 1989; Furuta et al., 2010), dorsal column nuclei (Giuffrida et al., 1986; Shin and Chapin, 1989), and spinal cord (Akintunde and Buxton, 1992).

However, each of the previous investigations has typically covered the projections of one or at most a few brain regions. To our knowledge, only one earlier investigation provided a brain-wide analysis of efferent projections from the S1 barrel cortex in mouse (Welker et al., 1988). Similar data are not available in rat, and no previous study has provided documentation of barrel cortex connectivity across the entire brain, allowing comparison of the projections originating from different S1 body representations. There is increasing awareness in the field about the need for comprehensive maps of rodent brain connectivity, and several large scale initiatives currently employ sophisticated axonal tracing paradigms and high-throughput methodologies to generate large amounts of experimental connectivity data from the mouse brain, such as the Allen Brain Atlas Mouse Connectivity project (www.brain-map.org) and the Mouse Connectome Project (www.mouseconnectome.org). These projects have made impressive amounts of image data from a large numbers of tract-tracing experiments publicly available, but few analyses of connectivity have yet been conducted with brain-wide coverage.

Efforts to aggregate information from the literature to gain overview of rat brain connectivity, such as the Brain Architecture Management System (BAMS, Bota et al., 2005, 2012) provide an overview of the major connections of S1. But the completeness of the presentations is difficult to assess due to lack of access to original data, and lack of brain-wide coverage in the original publications. A related question is whether neighboring body representations in S1 project to the same cortical and subcortical targets across the brain. Distinct topographical organization of S1 forelimb and whisker related projections to major target regions have been described (e.g., Brown et al., 1998; Hoover et al., 2003; Leergaard et al., 2000b), but differences in connectivity across the entire brain are largely unknown. Thus, beyond a few studies comparing S1 projections to different cortical areas (Hoffer et al., 2003) or corticostriatal, corticothalamic, and corticopontine projections from sensory and motor cortex (Hoffer et al., 2005), little is known about differences in densities and extent of S1 whisker barrel projections across all cortical and subcortical target regions. Such differences can only be assessed by brain-wide analyses of connectivity in the same experiments.

We here utilize an online resource containing high-resolution images with tract-tracing data (Zakiewicz et al., 2011; www.rbwb.org) to perform a brain-wide, semiquantitative analysis of the efferent connections of S1 barrel cortex. Our results allow comparison of the different well-known S1 efferent projections as well as less known projections to cortical and subcortical brain regions. We demonstrate distinct differences between S1 whisker and forelimb related projections and discuss possible functional implications of these findings. We finally compare our results to the overview of S1 connections provided by earlier publications and the BAMS database.

## Materials and methods

To determine the target regions of S1 efferent projections across the rat brain, we used a collection of high-resolution images of histological sections from six experiments in which axonal tracers were injected in whisker or forelimb representations in S1 (www.rbwb.org) (Zakiewicz et al., 2011).

Detailed procedures are described in Zakiewicz et al. (2011) and experimental metadata are available via the online data system (www.rbwb.org). All experimental procedures were approved by the institutional animal welfare committee of the University of Oslo and the Norwegian Animal Research Authority, and were in compliance with European Community regulations on animal well-being. Briefly, an anterograde axonal tracer (biotinylated dextran amine, BDA, or *Phaseolus vulgaris* leucoagglutinin, *Pha*-L), was injected in the cerebral cortex of anaesthetized adult Sprague Dawley or Wistar rats. After 7 days animals were sacrificed and transcardially perfused with 4% paraformaldehyde, and brains were removed for histological processing. 50 μm thick coronal sections were cut on a freezing microtome, and every second section was processed to visualize BDA or *Pha*-L (Gerfen and Sawchenko, 1984). Most sections were further counterstained with Thionine or Neutral red. Alternating sections through S1 were stained for cytochrome oxidase using the procedure of Wong-Riley et al. (Wong-Riley, 1979). High-resolution section images (TIFF format) were obtained through a 10× objective (Olympus UPlanApo, NA 0.40) using a motorized Olympus BX52 microscope running the Virtual Slide module of Neurolucida 7.0 (MBF Bioscience Inc., Williston, VT, USA). Images were converted to the Zoomify PFF format (Zoomify Inc., Santa Cruz, CA, USA) and assembled in an online data repository.

**Figure 1:**
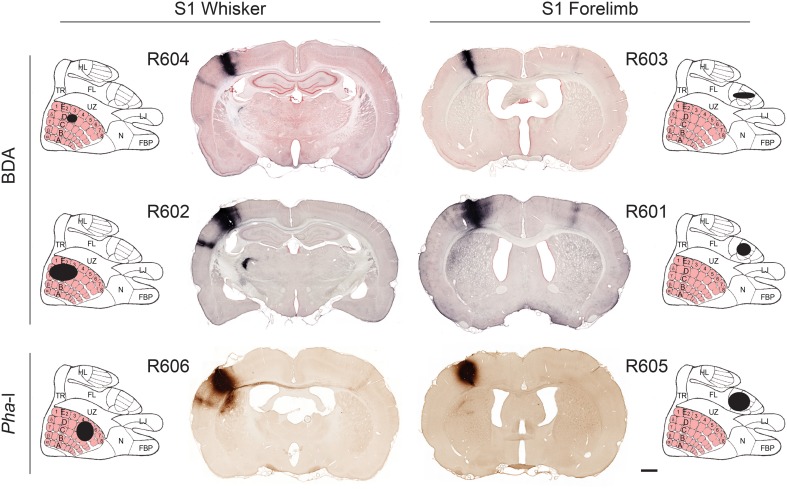
**Tracer injection sites**. Image of coronal sections from all cases investigated, showing BDA and *Pha*-L injection site centers in S1 whisker and forelimb representations. For each experiment, the estimated position and size of the injection sites is mapped onto a cartoon representation of the S1 cortex (redrawn from Chapin and Lin, 1984). The six injection sites cover the entire thickness of the cortex and vary in size (small to larger; **top** to **bottom** rows). Labeled axons are seen in most parts of S1. In the most of the coronal images shown, dense axonal labeling is distributed in columnar patterns across the entire cortical thickness of the ipsilateral and contralateral S2. Projections to the contralateral S2 are more prominent in the forelimb injected cases. Plexuses of labeled axons are also seen in the caudate-putamen complex and thalamus. Scale bar, 1 mm. FBP, furry buccal pad; FL, forelimb; HL, hindlimb; LJ, lower jaw; N, nose; TR, trunk; UZ, unresponsive zone.

The location of tracer injection sites were confirmed by analysis of anatomical landmarks and cytochrome oxidase staining pattern (Zakiewicz et al., 2011). All injections were columnar, and involved all cortical layers (Figure 1). To assess the size of the injection sites we used image analysis tools in Neurolucida. RGB images were converted (using the red or blue channel for sections stained with Neutral red or Thionine, respectively) to gray scale representations. The grayscale images were binarized with Neurolucida filters (Kodalith, fill holes, erode and pruning), and injection site volumes were estimated by summation of the measured areas multiplied with section spacing.

To identify target regions for efferent projections from the S1 whisker and forelimb representations, we systematically inspected all parts of the microscopic images from the six cases. Individual labeled axons were followed across sections to ensure that their targets were identified. The anatomical location of the observed labeling was determined by superimposing corresponding coronal atlas plates (Paxinos and Watson, 2007) to each image, using affine transformations applied in Adobe Illustrator CS5 (Adobe Systems Inc, San Jose, CA, USA). For each region the spatial registration of atlas overlay was adjusted on the basis of local landmarks and cytoarchitectonic patterns. The nomenclature and abbreviations used in this report are adopted from Paxinos and Watson (2007).

The amount of labeled fibers in each anatomical (sub) region was semiquantitatively assessed by a single examiner, scoring the observed labeling using a density rating system using predefined criteria. The labeling was scored as “weak” (score = 1) for a few labeled fibers that were possible to count, as “moderate” (score = 2) for several fibers that could be individually discerned but not readily counted, and as “strong” (score = 3) for many labeled fibers forming dense plexuses where individual fibers could not be discerned.

For comparison with connectivity reports registered in the BAMS_2_ database (http://brancusi1.usc.edu/) we used the online query tools of this database, supported with customized data files kindly provided by Dr. Mihail Bota (personal communication). The connections reported in the BAMS_2_ are based on terminology used in the Swanson (1998) atlas of the rat brain. At the detail level of the target regions reported here, this terminology is compatible with the Paxinos and Watson (2007). Strengths indicated for connections in BAMS_2_ were re-interpreted to match that of the present study, aided by the collator notes registered in BAMS_2_, and cross-check with original references. The annotation of strength in BAMS_2_ had a higher granularity and was reinterpreted to our semiquantitative scale as follows, scores “very light” and “light” were interpreted as light; “light/moderate” and “moderate” as moderate, and “moderate/strong,” “strong,” and “very strong” as strong.

## Results

To identify the cortical and subcortical brain regions receiving projections from the rat S1 whisker barrel cortex, and to compare the projections of S1 whisker representations to the neighboring S1 forelimb representation, we have examined the distribution of anterogradely labeled axons arising from axonal tracer injections in S1 whisker or forelimb representations in a collection of section images from six experiments (www.rbwb.org; Zakiewicz et al., 2011).

### General features of labeling

The six injection sites varied in volume (0.23–2.97 mm^3^; Table 1), but had sharp boundaries and covered the entire thickness of the cerebral cortex, without involvement of the underlying white matter (Figure 1). The positions of the injection site centers were inferred from histological analyses of anatomical landmarks and cytochrome oxidase staining patterns (Zakiewicz et al., 2011). Inspection of sections stained for cytochrome oxidase revealed that the injections into S1 barrel cortex involved both barrels (D2, D3 or D5) and adjacent septa.

**Table 1:**
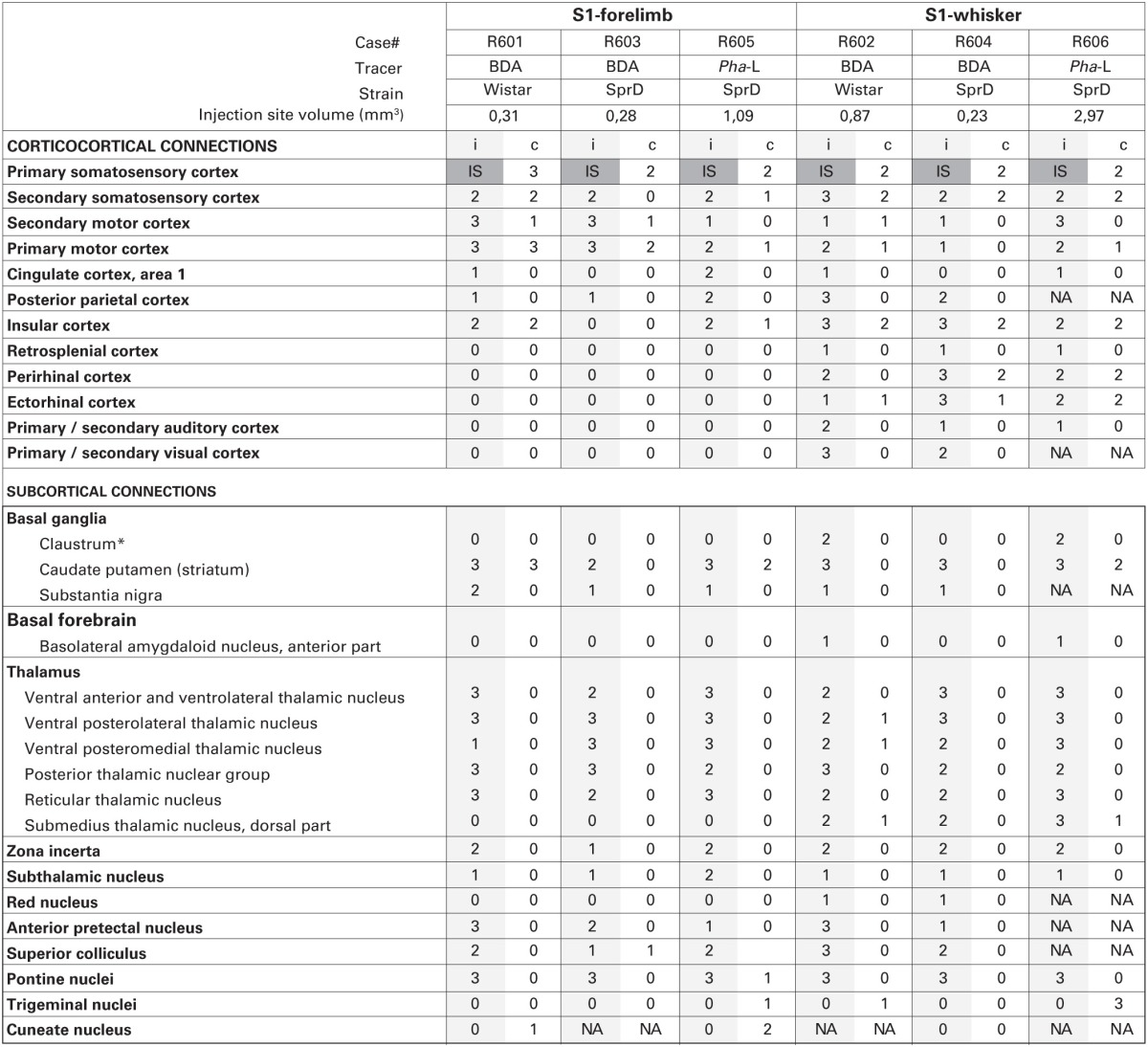
**Summary of observations and semiquantitative assessment**.

IS, injection site; NA, not available.

Semiquantitative assessment of amount of labeled fibers:

0, confirmed absence of labeled fibers.

1, weak; few fibers that are possible to count.

2, modest; several fibers that can be individually discerned but not readily counted.

3, strong; many fibers forming dense plexuses where individual fibers cannot be discerned.

^*^Anatomical location was assigned according to Paxinos and Watson (2007). Note that labeled fibers were observed in anterior regions underlying the forceps minor of the corpus callossum, >3 mm anterior of bregma, and that recent proteomic analyses (Mathur et al., 2009) indicate that this part of the brain should not be included in the structural definition of the claustrum.

**Figure 2:**
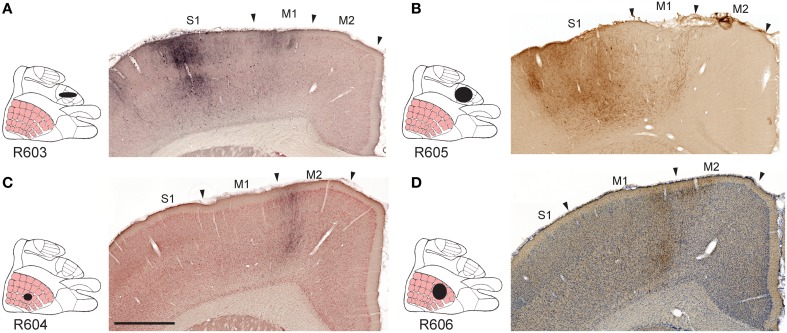
**Examples of projections to motor cortex**. Images exemplifying S1 projections to the ipsilateral primary and secondary motor cortex. **(A–D)**, Labeling in M1 and M2 originating from S1 forelimb **(A,B)** and S1 whisker **(C,D)** representations, distributed in distinct columns in M1, and partly across the boundary between M1 and M2. M1, primary motor cortex; M2, secondary motor cortex; S1, primary somatosensory cortex. Scale bar, 0.5 mm.

**Figure 3:**
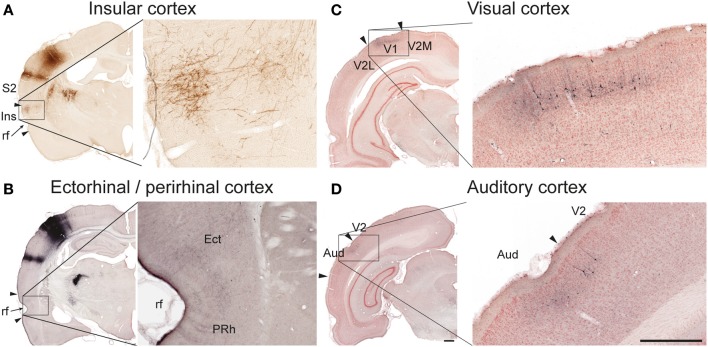
**Examples of projections to temporal and occipital cortex**. In the insular (**A**, case R606) and ectorhinal (**B**, case R602) cortex, labeling is primarily distributed in superficial cortical layers. In the primary visual (**C**, case R602) and auditory (**D**, case R602) cortices both labeled axons and retrogradely labeled neurons are observed in superficial layers. Aud, auditory cortex; Ect, ectorhinal cortex; Ins, insular cortex; PRh, perirhinal cortex; rf, rhinal fissure; S2, secondary somatosensory cortex; V1 primary visual cortex, V2, secondary visual cortex, V2M, secondary visual cortex, medial area; V2L, secondary visual cortex, lateral area. Scale bars, 0.5 mm.

**Figure 4:**
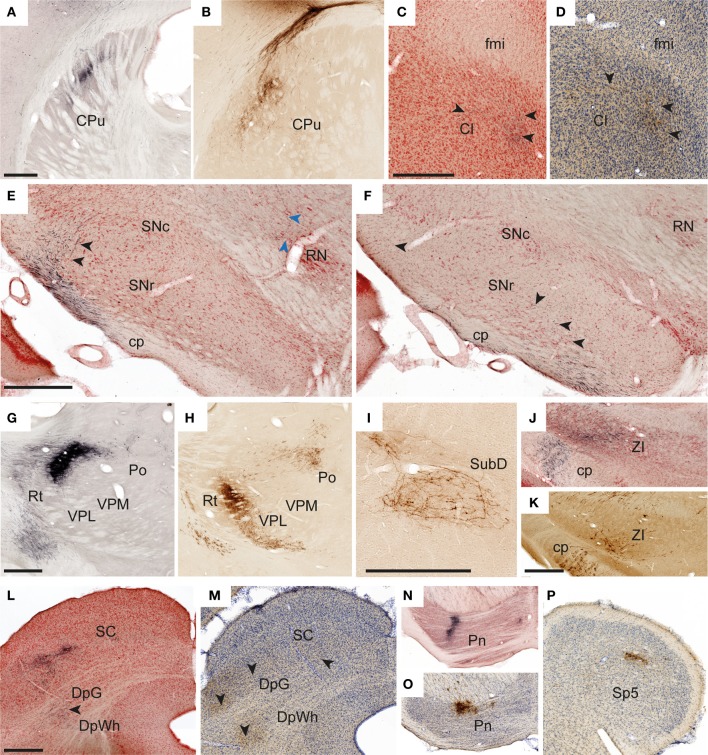
**Examples of subcortical labeling**. Images illustrating observed axonal labeling in a selection of subcortical regions. **(A,B)** Elongated plexuses of labeling in the dorsal striatum, arising from S1 whisker (**A**, case R602) and forelimb (**B**, case R605) representations. **(C,D)** Labeled fibers in anterior parts of the claustrum as defined in the employed atlas (Paxinos and Watson, 2007), but in a location that according to recent proteomic analysis is not part of the claustrum (Mathur et al., 2009). (**C**, case R602; **D**, case R606). **(E,F)** Widespread labeled fibers in the lateral, reticular part of the substantia nigra (**E**, case R602; **F**, case R601). Black arrowheads in **(E,F)** indicate labeled fibers in the substantita nigra. Blue arrowheads in **E** indicate a labeled fiber reaching the red nucleus. **(G,H)** Sharply defined dense plexuses of labeling in the thalamus (**G**, case R602; **H**, case R605). **(I)** Example of a loosely organized plexus of labeled fibers in the submedius nucleus thalamus (case R606). **(J,K)** Labeled fibers in the zona incerta (**J**, case R602M; **K**, case R605). **(L,M)** Examples showing labeled fibers in the superficial and deep layers of the superior colliculus (**L**, case R602; **M**, case R605). **(N,O)** Dense plexuses of labeling in the pontine nuclei (**N**, case R602; **O**, case R605). **(P)** Discrete labeling in the caudal part of the contralateral spinal trigeminal nucleus (R602) Cl, claustrum; cp, cerebral peduncle; CPu, caudate putamen (striatum); DpG, deep gray layer of the superior colliculus; DpWh, deep white layer of the superior colliculus; fmi, forceps minor of the corpus callosum; Pn, pontine nuclei; Po, posterior thalamic nuclear group; RN, red nucleus; Rt, reticular thalamic nucleus; SC, superior colliculus; SNc, substantia nigra, compact part; SNr, substantia nigra, reticular part; Sp5, spinal trigeminal nucleus; SubD, submedius nucleus thalamus, dorsal part; VPL, ventral posterolateral thalamic nucleus; VPM, ventral posteromedial thalamic nucleus; ZI, zona incerta. Scale bars, 0.5 mm.

The two tracers (BDA and *Pha*-L) both gave rise to distinctly labeled axons in intracortical and subcortical targets (Figures 1–4, summarized in Tables 1, 2). The fibers where sharply defined with visible beaded varicosities, readily observed in the high-resolution images shown in the Whole Brain Connectivity Atlas. Retrogradely labeled cells were also observed in several regions in cases injected with BDA. This labeling is commented on below, but not included in our semiquantitative analysis due to the less robust properties of the 10 kDa BDA tracer for retrograde tracing (Lanciego and Wouterlood, 2000, 2011). Cyto- and chemoarchitectural features were helpful to determine anatomical boundaries. The shape, size, and density of labeled fibers were highly similar across cases, although the amount of labeling reflected the size of the injection sites. While several bilateral projections were observed, the amount of contralateral labeling was always lower, and tended to be distributed in a pattern mirroring the ipsilateral labeling.

**Table 2:**
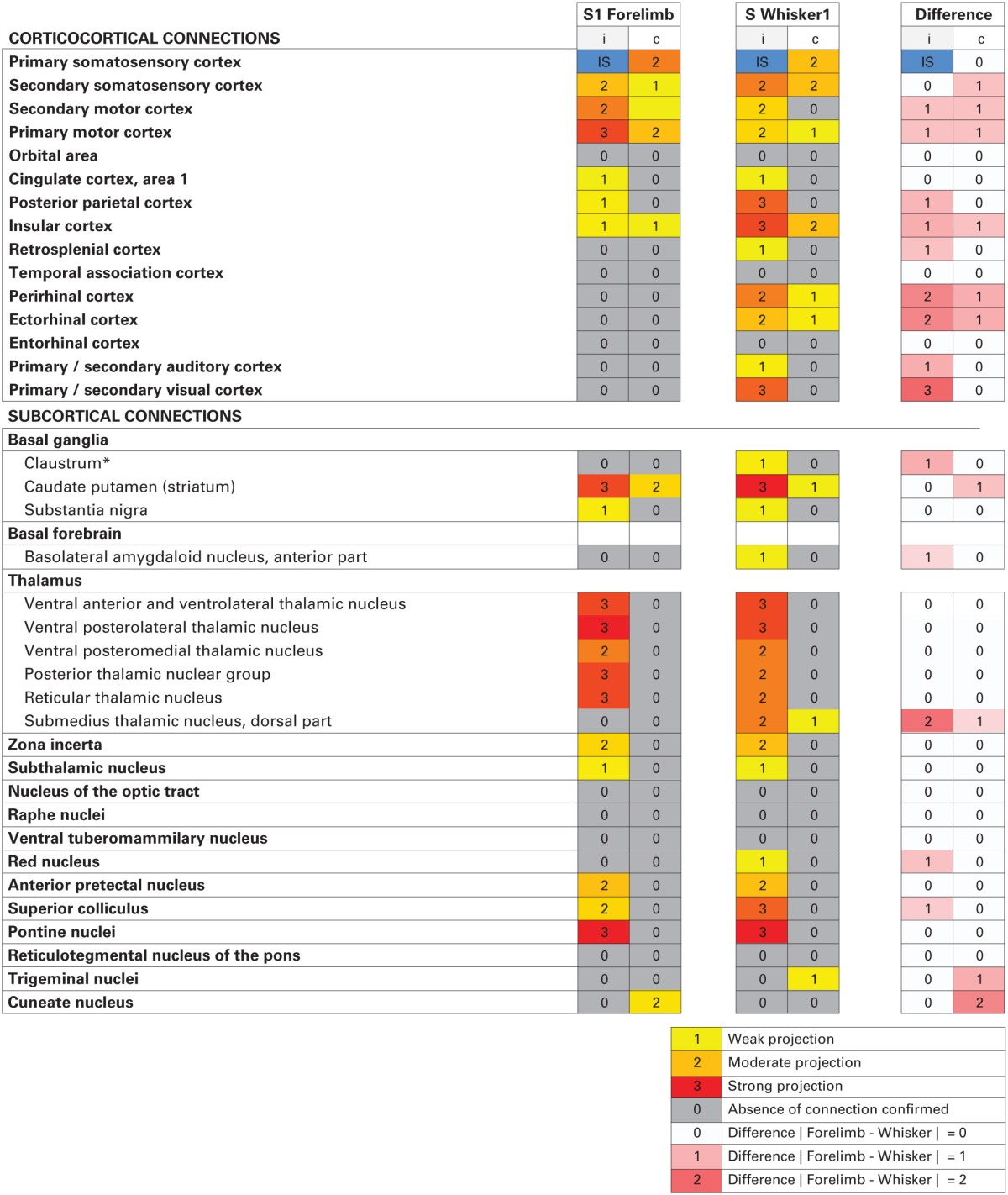
**Overview of S1 efferent projections**.

Columns 1 (S1 forelimb) and 2 (S1 whisker) show average projections (with color-coded strength) observed in the present study, cumulated from all six cases (Table 1). Column 3 shows the difference between the semiquantitative projection scores from S1 whisker and forelimb representations, such that 0 indicates no difference, while numbers 1–3 indicate degrees of difference.

### Cortico-cortical projections

#### Motor and somatosensory cortex

All tracer injections gave rise to labeled axons in most parts of the injected S1 cortex, reflecting the well-known intrinsic connectivity of S1 (Fabri and Burton, 1991a). We found substantial amounts of labeled fibers in the contralateral S1, bilaterally in the primary motor cortex (M1) and the secondary somatosensory cortex (S2), and to a lesser extent the secondary motor cortex (M2; Figures 2A–D), in agreement with earlier observations (Donoghue and Parham, 1983; Reep et al., 1990; Fabri and Burton, 1991a; Wright et al., 1999; Hoffer et al., 2003; Alloway et al., 2004, 2008; Hoffer et al., 2005; Colechio and Alloway, 2009; Smith and Alloway, 2013). The amount of forelimb related projections to motor areas was consistently higher than whisker related projections, relative to the size of the injection sites (Table 1). It should be noted that the region drawn as M2 in the employed reference atlas (Paxinos and Watson, 2007) includes the medial and lateral agranular cortex (Donoghue and Wise, 1982). Indeed, the labeling observed in our material (Figures 2C,D) fits well with the S1 projections recently described to distribute across the transition zone between the medial and lateral agranular cortex (Smith and Alloway, 2013).

#### Insular and posterior parietal cortex

In five of six cases, we found significant amounts of labeled axons distributed bilaterally in the insular cortex (Table 1; Figure 3A), in agreement with earlier descriptions of S1 projections to the parietal ventral cortex (Fabri and Burton, 1991a), which corresponds to the insular cortex as delineated in the reference atlas (Paxinos and Watson, 2007). In the three cases injected in the barrel cortex, we also found labeling bilaterally in the perirhinal and ectorhinal cortex (Figure 3B), in line with earlier studies (Fabri and Burton, 1991a; Naber et al., 2000).

Further, in the two S1 whisker experiments we observed substantial labeling in the posterior parietal cortex, in agreement with earlier reports (Koralek et al., 1990; Fabri and Burton, 1991a; Lee et al., 2011). In the three S1 forelimb experiments moderate amounts of labeling were found in the posterior parietal cortex.

#### Cingulate and retrospleninal cortex

In the four cases with the largest injection sites we observed some labeling in area 1 of the ipsilateral cingulate cortex. In the cases injected in the S1 barrel cortex a modest amount of labeling was also seen in the ipsilateral retrosplenial cortex. Our observations confirm earlier reports of moderate or weak projections from S1 to the anterior cingulate cortex (Reep et al., 1990; Van Eden et al., 1992; Condé et al., 1995) and retrosplenial cortex (Shibata and Naito, 2008).

#### Visual and auditory cortex

In the two animals receiving BDA injections in the S1 barrel cortex, discrete patches of labeled fibers and considerable numbers of retrogradely labeled neurons were observed in the ipsilateral primary and secondary visual cortex (Figure 3C), as well as in the neighboring auditory cortex (Figure 3D), confirming earlier findings by electrophysiology and tract tracing (Frostig et al., 2008; Sieben et al., 2013).

### Subcortical projections

#### Basal ganglia

In all experiments, dense, elongated clusters of labeled axons were seen in the ipsilateral dorsal striatum (Figures 4A,B), and in some we also found smaller amounts of labeling in mirrored locations in the contralateral striatum. The corticostriatal projections from the S1 barrel region are well known, and the somatotopic arrangement of projections from different body representations is well characterized (Brown et al., 1998; Alloway et al., 1999; Hoffer and Alloway, 2001).

We further observed weak projections to other parts of the basal ganglia. In two cases (R605 and R606), a few individual labeled fibers were observed in the amygdalostriatal transition area of the ventral striatum, which presumably were en route to the basolateral amygdaloid nucleus (see below). In two experiments with relatively large BDA or Pha-L injection sites in the S1 barrel cortex, labeled axons were visible in anterior parts of the ipsilateral claustrum, in the region located ventrally to the forceps minor of the corpus callosum, >3 mm anterior of bregma (Figure 4C). It was earlier demonstrated by retrograde tracing that this region projects to S1 (Zhang and WDeschenes, 1998). Our findings of (anterograde) Pha-L labeling here thus indicate direct projections from S1 whisker representations. However, a recent study (Smith et al., 2012) failed to demonstrate corticoclaustral projections from S1 whisker representations, at least at more posterior levels. Recent proteomic analyses indicate that the claustrum is limited anteriorly to coronal levels which include the striatum (Mathur et al., 2009), and not the anterior region underlying the forceps minor of the corpus callosum, where we observed labeling. This suggests that the labeling we observed in the region defined as claustrum in our reference atlas (Paxinos and Watson, 2007), should not be interpreted as corticoclaustral projections (see footnote to Table 1).

Finally, in all cases but one (in which relevant sections were missing) some widespread labeled fibers were found in the ipsilateral reticular part of the substantia nigra (Figures 4E,F). While corticonigral projections from prefrontal and motor areas have been reported earlier (Gerfen et al., 1982), evidence of S1 corticonigral projections has to our knowledge not been reported before.

#### Basal forebrain

While the basal forebrain is known to project to the cerebral cortex (Sripanidkulchai et al., 1984), it is less clear if the basal forebrain receives projections from S1. In two cases injected in S1 whisker representations, we observed a few labeled fibers in the anterior part of the basolateral amygdaloid nucleus.

#### Thalamus

In agreement with earlier reports (Staiger et al., 1999; Wright et al., 1999; Veinante et al., 2000; Wright et al., 2000) we found substantial ipsilateral projections to the ventral posterolateral and ventral posteromedial thalamic nuclei, the posterior thalamic nuclear group, and reticular thalamic nucleus (Figures 4G,H). Also, in all animals injected with BDA, multiple retrogradely labeled neurons were observed in these regions, reflecting the well-known reciprocal connections between S1 and the thalamus (Saporta and Kruger, 1977; Koralek et al., 1988; Berendse and Groenewegen, 1991; Fabri and Burton, 1991a). Further, in the three experiments involving the S1 whisker barrel cortex, we also found a substantial labeling in the dorsal part of the ipsilateral submedius thalamic nucleus (Figure 4I), which in the two cases with the largest injection sites also included some contralateral labeling. The submedius nucleus is known to receive nociceptive input from the trigeminal nuclei and spinal cord, and has been implicated in modulatory nociceptive processes (Craig and Burton, 1981; Dostrovsky and Guilbaud, 1988; Miletic and Coffield, 1989). This region is reciprocally connected with the cerebral cortex in cat (Craig et al., 1982), but these connections have, as far as we can determine, not been emphasized in earlier studies of the rat brain.

#### Zona incerta, subthalamic nucleus, and red nucleus

Moderate amounts of fibers were found in the ipsilateral zona incerta (Figures 4J,K) and subthalamic nucleus, in line with earlier observations (Rouzaire-Dubois and Scarnati, 1985; Nicolelis et al., 1992). In cases injected into the whisker barrel cortex we also found a few labeled fibers in the red nucleus (Figure 4E). Although somatosensory projections to the red nucleus have been described by use of electrophysiological recordings (Ebrahimi-Gaillard and Roger, 1993) and retrograde tracing technique (Bernays et al., 1988; Akintunde and Buxton, 1992), our anterograde tracing results indicate that corticorubral projections from S1 forelimb and whisker representations are rather insignificant.

#### Anterior pretectal nucleus and superior colliculus

In all cases but one (from which relevant material was missing), moderate amounts of labeled fibers were observed in the anterior pretectal nucleus and superior colliculus. In the anterior pretectal nucleus loose plexuses of labeled fibers are seen, confirming earlier observations of sparse connections by means of retrograde tracing (Cadussea and Roger, 1991). In the superior colliculus (Figures 4L,M), labeled fibers were loosely distributed across several layers, and did not aggregate in distinct, topographically organized clusters as described in several earlier studies (Wise and Jones, 1977b; Schwarz and Their, 1995; Hoffer et al., 2003, 2005).

#### Pontine nuclei

In all cases, we observed strong projections to the ipsilateral pontine nuclei (Figures 4N,O). These fibers were distributed in several well defined clusters in agreement with earlier observations (Leergaard, 2003; Leergaard and Bjaalie, 2007).

#### Trigeminal nuclei

Although the trigeminal nuclei are known to receive significant projections from the contralateral S1 (Wise et al., 1979; Killackey et al., 1989; Furuta et al., 2010; Tomita et al., 2012), we only observed limited amounts of labeled fibers in the trigeminal nuclei (Figure 4P) in three of six cases, including two cases with tracer injection into the S1 whisker representation (R602, R606) and one case with tracer injection in the forelimb representation (R605). These were the three experiments with relatively large injection sites (Table 1). The modest labeling observed in our material stands in contrast to the rather abundant corticotrigeminal labeling seen after tracer injections into S1 orofacial regions (Tomita et al., 2012).

#### Dorsal column nuclei and spinal cord

The corticocuneate and corticospinal projections of S1 are known (Wise and Jones, 1977a; Lue et al., 1997; Martinez-Lorenzana et al., 2001) and we also observed substantial amounts of labeled fibers in the corticobulbar and corticospinal tracts, which in some cases (where material was available) could be followed to the contralateral dorsal corticospinal tract. Sparse amounts of labeled fibers were observed in contralateral cuneate nuclei in two experiments where tracer was injected into the S1 forelimb representation, but it should be noted that in three cases material was not available from this region. The amount of labeling observed in our material is compatible with the observation that cortical neurons, retrogradely labeled by tracer deposits in the dorsal column nuclei, are relatively widespread in S1 (Martinez-Lorenzana et al., 2001).

### Negative findings in brain regions otherwise not mentioned

The present analysis covered all sections present in the brain-wide collection of section images available in the Whole Brain Connectivity Atlas. All regions and subregions of the brain were manually inspected for labeling. Thus, our results strongly indicate absence of projections from S1 whisker and forelimb representations to brain regions not included in Table 1.

### Comparison of efferent projections from S1 forelimb and whisker representations

**Figure 5:**
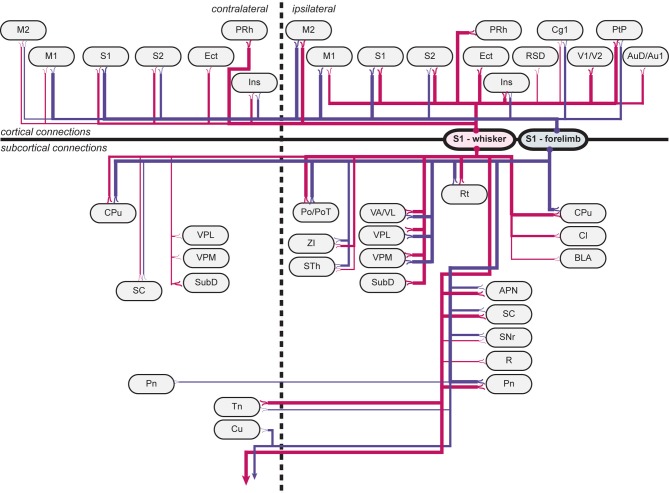
**Wiring diagram, summarizing findings**. Summary diagram showing all connections observed in experiments. connections arising from s1 whisker representations are indicated by red lines, and connections arising from s1 forelimb representations are indicated by blue lines. line thickness corresponds to the amount of labeling (low, medium or high) observed, as indicated in Table 1. apn, anterior pretectal nucleus; au1, primary auditory cortex; aud, secondary auditory cortex, dorsal area; bla, basolateral amygdaloid nucleus, anterior part; cg1, cingulate cortex, area 1; cl, claustrum; cpu, caudate putamen (striatum); cu, cuneate nucleus; ect, ectorhinal cortex; ins, insular cortex; m1, primary motor cortex; m2, secondary motor cortex; pn, pontine nuclei; po, posterior thalamic nuclear group; pot, posterior thalamic nuclear group, triangular part; prh, perirhinal cortex; ptp, posterior parietal cortex; r, red nucleus; rsd, retrosplenial cortex; rt, reticular thalamic nucleus; s1, primary somatosensory cortex; s2, secondary somatosensory cortex; sc, superior colliculus; snr, substantia nigra, reticular part; sth, subthalamic nucleus; subd, submedius thalamic nucleus, dorsal part; tn trigeminal nuclei; v1, primary visual cortex; v2, secondary visual cortex; va/vl, ventral anterior and ventrolateral thalamic nucleus; vpl, ventral posterolateral thalamic nucleus; vpm, ventral posteromedial thalamic nucleus; zi, zona incerta.

Overall, our results show that S1 forelimb and whisker projections target many of the same cortical and subcortical regions (Tables 1, 2; Figure 5), although with different topographical distributions within each region. Some important differences were observed (Table 2): The S1 whisker barrel cortex projects to several cortical areas which do not receive projections from the S1 forelimb region, such as the retrosplenial cortex, perirhinal, ectorhinal, auditory, and visual cortex, S1 forelimb representations have more prominent projections to the motor areas (M1 and M2), and projections from S1 whisker barrel to insular cortex are more abundant. We further observed some differences in the subcortical projections: the S1 barrel cortex targets the submedius thalamic nucleus, provides stronger projections to the superior colliculus and trigeminal nuclei, has weak projections to the basolateral amygdaloid nucleus and red nucleus, but no projections to the cuneate nucleus.

### Comparison with accumulated legacy data

A large number of previous investigations have explored the connections of the S1 barrel cortex (see references above, and review by Bosman et al., 2011). Many publications on rat brain connections have also been collated and registered in the BAMS_2_ database (http://brancusi1.usc.edu/), although coverage here is far from exhaustive. We compared our results with S1 efferent connections registered in BAMS_2_ (only ipsilateral data were available), connections mentioned in a recent review article (Bosman et al., 2011), and projections reported in an earlier brain-wide tract-tracing study conducted in mice (Welker et al., 1988). Table 3 provides an overview of these comparisons.

**Table 3:**
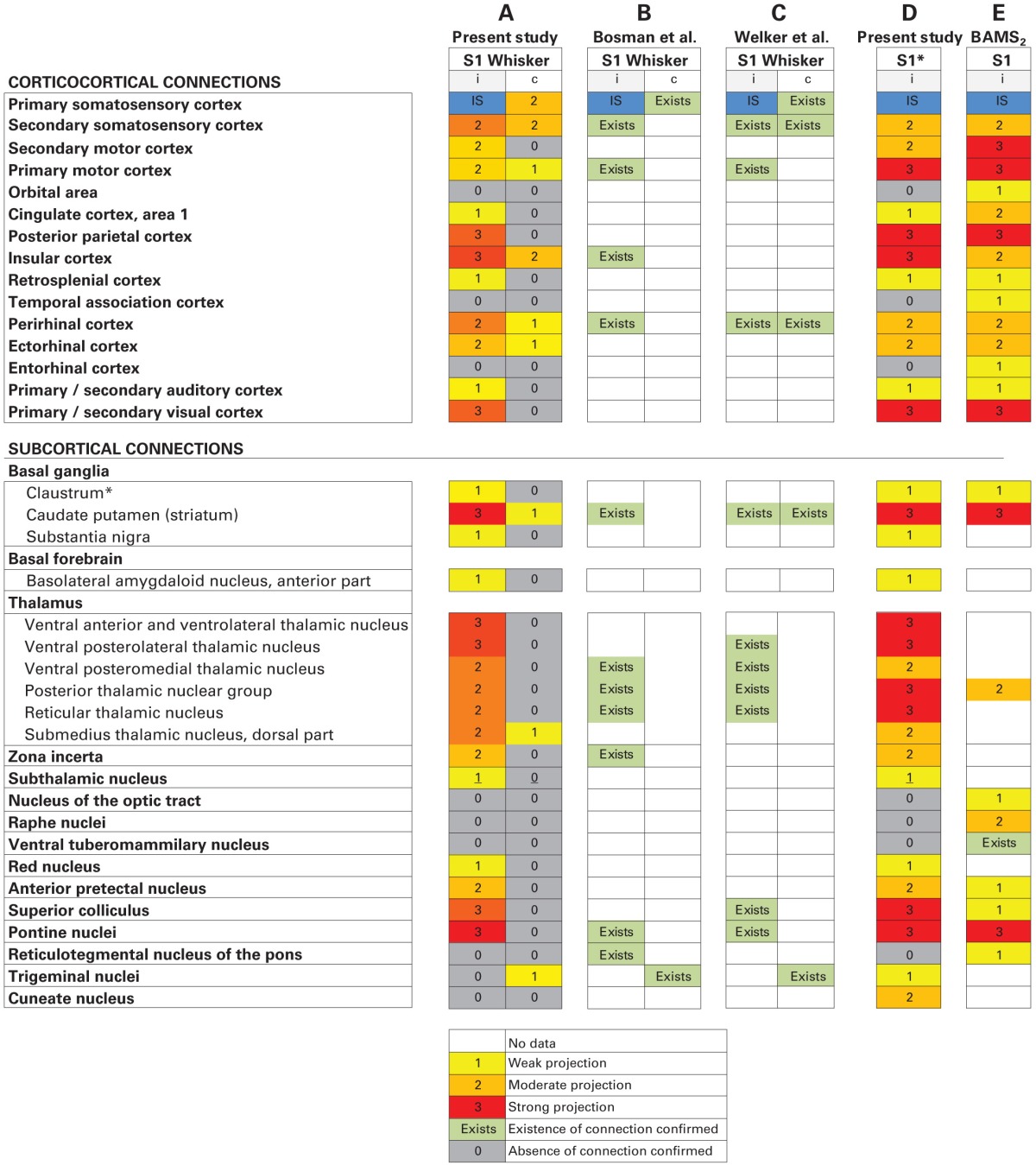
**Comparison with legacy data**.

Columns A–C show a comparison of efferent projections from the S1 barrel cortex reported in the present study (column A), a recent review report of the rodent barrel cortex (Bosman et al., 2011), and a brain-wide analysis in mice (Welker et al., 1988). Columns D and E provide a comparison of our findings with ipsilateral projections of the entire S1 region registered in the BAMS_ıt 2_ database, with projection strength re-interpreted to the scale employed in the present study. Data in column D show the maximum average S1 forelimb or whisker projections (from Table 2).

Comparing our results with BAMS_2_ (Table 2), we find that all ipsilateral cortico-cortical projections observed in our analysis are registered in BAMS_2_ with corresponding strengths, and further that BAMS_2_ contains reports of some additional weak projections to the orbital area (Paperna and Malach, 1991; Reep et al., 1996), temporal association cortex (Paperna and Malach, 1991), and entorhinal cortex (Burwell and Amaral, 1998), regions in which we found no labeling. Consulting the original reports we find that these weak connections were identified by observation of few scattered neurons retrogradely labeled by tracer injection in the different target regions (see, e.g., Burwell and Amaral, 1998). It is unclear whether such cells were located in S1 whisker or forelimb representations.

The collection of subcortical connections of S1 registered in BAMS_2_ is, however, different from our account, as major corticothalamic projections are not included in BAMS_2_. The annotated strength of S1 projections to the striatum, posterior thalamic nuclear group, anterior pretectal nucleus, superior colliculus, and pontine nuclei registered matched fairly well with our results. Projections to zona incerta, subthalamic nucleus, red nucleus, trigeminal nuclei, and cuneate nucleus are so far not included in BAMS_2_. BAMS_2_ contained reports of weak subcortical projections to the nucleus of the optic tract (Schmidt et al., 1993), raphe nuclei (O'Hearn and Molliver, 1984), ventral tuberomammilary nucleus (Köhler et al., 1985), and reticulotegmental nucleus of the pons (O'Hearn and Molliver, 1984), all regions in which we found no labeling. Consulting the original articles, we find that these concern retrograde tracing experiments, yielding some labeling in the parietal cortex, which may or may not include the regions investigated in the present study.

Finally, when comparing our results with a recent review of the rodent barrel cortex (Bosman et al., 2011) and an earlier brain-wide tract tracing study in the mouse brain (Welker et al., 1988), we find that all major connections are mentioned in these reports, while most of the moderate or weaker projections observed in our study (and to some extent also registered in BAMS_2_) are not included.

## Discussion

We have mapped projections to cortical and subcortical targets originating from the S1 whisker and forelimb representations in rat. Anterogradely labeled axons, originating from tracer injections in S1 cortex of six animals, were identified across a large collection of histological image (Zakiewicz et al., 2011). Compared to earlier efforts, our brain-wide analysis (summarized in Tables 1–3, and Figure 5) contributes more complete and detailed information about S1 efferent projections, both regarding completeness and information about differences between projections from the S1 whisker and forelimb cortex. Our comparison of the efferent projections of S1 whisker and forelimb representations shows that these generally reach the same targets, but that projections from the S1 barrel cortex target more (sensory related) cortical areas as well as some additional subcortical brain regions. The present analysis is based on experimental tract tracing data from adult male Sprague Dawley and Wistar rats, using two different axonal tracers (Table 1). *Pha*-L is considered to be a pure anterograde tracer showing little uptake by fibers of passage (Wouterlood and Jorritsma-Byham, 1993), while BDA can be taken up by passing fibers and also has retrograde properties which may give rise to secondary, or indirect, anterograde labeling (Merchan et al., 1994; Merchan and Berbel, 1996; Lanciego and Wouterlood, 2000). Regardless of these different parameters, the overall pattern of connections observed in this material is remarkably consistent across strain and tracers used (Table 1).

All six injection sites were columnar of shape and involved all cortical layers without infringement of white matter in the external capsule. The experiments provide information about the efferent connectivity of the entire S1 injection sites, but without possibility to differentiate layer-specific connections. With semiquantitative assessment we observe a robust relationship between injection site volumes and amount of labeling. The relatively small injection sites may account for weak projections. Hence, absence of labeled fibers in the cingulate cortex, claustrum, basolateral amygdaloid nucleus, and trigeminal nuclei in one (case R604) out of three experiments with tracer injection in the S1 barrel cortex, can be explained by the considerably smaller size of the BDA injection.

Nearly all of the connections demonstrated in our survey have been reported earlier, and only a few projections not observed in our material have been reported in the literature. Thus, our report is in general agreement with earlier literature, and provides the so far most complete overview of the efferent projections of rat S1 barrel cortex. However, an overwhelming wealth of scientific reports describing various details reflecting the connectivity of the S1 barrel cortex exists, and a comprehensive review of S1 connectivity literature is beyond the scope of our study.

Discrepancies with earlier observations may reflect biological variability or variation in the employed tract tracing paradigms (tracer properties, size and position of tracer injection site, and effective zone of tracer uptake). Reports of connections not observed in the present study mainly concerns retrograde tracing studies demonstrating sparse amounts of labeled neurons in the parietal cortex, which may or may not involve the specific S1 representations investigated in our study. There is also a concern that some connections identified by retrograde tracing may involve false positive labeling caused by contamination or uptake of tracer in passing fibers. Our results further highlight the challenges related to the use of different nomenclature and boundary definitions, and the need for efficient ways to compare and translate between different brain atlases. This is particularly evident with respect to the claustrum, where the employed atlas (Paxinos and Watson, 2007) does not hold more recent structural information (Mathur et al., 2009). Interestingly, it is thus unclear which anatomical location would be appropriate for the fibers observed in the anterior part of the region previously known as the anterior part of the claustrum. A related problem is found with our observations of S1 barrel cortex projections to the ectorhinal cortex, which is referred to by different terms (postrhinal cortex) in earlier studies of connections (Burwell et al., 1995; Naber et al., 2000).

Some more subtle differences between our results and earlier reports should be mentioned: The observed S1 projections to the red nucleus appear very weak in our material, which is at odds with earlier electrophysiological reports of somatosensory cortical influence of the red nucleus (Ebrahimi-Gaillard and Roger, 1993). This discrepancy may reflect the selection of S1 representations involved in our study. Similarly, the projections to the superior colliculus are unexpectedly weak in our material, as compared to other investigations which have reported strong corticotectal projections from the S1 barrel cortex (Schwarz and Their, 1995; Hoffer et al., 2003, 2005). We have no explanation for this difference, other than experimental factors such as the size and position of the tracer injections.

Overall, relative to S1 forelimb representation, our study shows that the S1 whisker barrel cortex has more abundant projections to cortical and subcortical regions that are relevant in context of sensory exploration, such as the perirhinal and ectorhinal cortex which are implicated in sensory integration and gating (Naber et al., 2000; Rodgers et al., 2008), and to the submedius nucleus of the thalamus which modulates nociceptive processes (Craig and Burton, 1981; Miletic and Coffield, 1989; Blomqvist et al., 1992).

The presented results are of relevance for ongoing large-scale efforts to systematically map connections in the rodent brain, such as the Mouse Brain Connectome Project and the Allen Mouse Brain Connectivity Atlas. These initiatives provide access to very large collections of images containing tract-tracing data resulting from tracer injections in various parts of the mouse brain. Similar to the Whole Brain Connectivity Atlas resource utilized in our project, these projects provide online access to serial image data in web browsers, allowing investigators to inspect tracer injection sites and ensuing labeling patterns. These resources are conceptually quite similar to the data collection investigated in the present study, and face the same challenges with respect to analysis, interpretation, and extraction of knowledge about connectivity. The three-dimensional image viewer provided by the Allen Mouse Brain Connectivity Atlas offers additional advantages. When looking up experiments involving the S1 barrel cortex, it is straightforward to view well-known projections to e.g., the ipsilateral M1, contralateral S1, striatum, thalamus, and pontine nuclei. But existence of projections to other known targets can only be confirmed by more detailed anatomical analysis of individual section images.

## Conclusions

We have performed the first brain-wide survey of whisker and forelimb related S1 efferent connections in rat based on data shared through an online atlas. The observed connectivity patterns were highly consistent across the 6 experiments, and some distinct differences were observed between projections from S1 forelimb and whisker representations. In comparison to earlier efforts to generate overviews of S1 efferent projections in the rodent brain based on the available literature, our analysis has provided a more detailed overview, allowing assessment of projection strength across target regions and comparison of projections originating from different subregions of S1. Access to organized collections of raw image data and accompanying tools for viewing and inspection of the data represents a first step only. Conclusions regarding connectivity require attention to interpretation of location of labeling in relation to boundaries and potential sources of error in the experiments. Our study sheds light on important challenges inherent to such analyses.

### Conflict of interest statement

The authors declare that the research was conducted in the absence of any commercial or financial relationships that could be construed as a potential conflict of interest.
